# Again/st the Mad Subject ^1^

**DOI:** 10.1111/nup.70079

**Published:** 2026-04-08

**Authors:** Simon Adam

**Affiliations:** ^1^ York University Toronto Ontario Canada

**Keywords:** becoming‐imperceptible, escape, identity, madness, ontology, posthumanism, subjectivity

## Abstract

Although widely contested, psychiatry continues to function as a dominant governing framework for mental distress within contemporary healthcare. While critiques of psychiatry have proliferated across survivor movements, mad studies, neurodiversity advocacy, and critical nursing scholarship, much of this work remains tethered to humanist assumptions that seek recognition, inclusion, or rights for a stabilized subject. This article argues that psychiatry functions not merely as a contested medical specialty but as a structural determinant of harm that systematically produces hierarchies of the human through diagnostic legibility, coercive care, and moralized governance. Focusing on mental health nursing, the article examines how nursing knowledge and practice are entangled with psychiatric classification, risk frameworks, and legislative authority, implicating the discipline in the reproduction of psychiatric power even when care is benevolently and ethically motivated. Rather than positioning psychiatry as simply helpful or harmful, the analysis reframes it as a humanist apparatus of capture that stabilizes subjects in order to render them governable. While identity‐based critiques and activist movements have achieved crucial reforms, this article contends that such strategies risk reinscribing the very ontological conditions psychiatry requires to persist. Drawing on posthuman and poststructural theory, the article advances an alternative orientation for nursing grounded in becoming, imperceptibility, and relational ontology. Madness is theorized not as an identity to be recognized, but as a destabilizing force that exposes the limits of the humanist subject. The article concludes by proposing posthuman figurations of nursing care that move beyond subject‐centeredness toward practices attentive to assemblages, processes, and lines of flight–forms of care that do not oppose psychiatry directly but render it increasingly irrelevant by exceeding its capacity for capture.

A hauntology of the dispossessed, the excluded, and those violently excommunicated from the Western ethical order of the ‘human’ remains the most pervasive psychological feature of imperial power.

Arthur Kroker

## 1 Introduction: Psychiatry as a Structural Determinant of Harm

Psychiatry occupies a paradoxical position within contemporary healthcare. As one of the most powerful modern institutions governing mental distress, it exerts profound influence over clinical practice, policy, and public understandings of madness. At the same time, psychiatry has been the object of sustained and escalating critique, with scholars, activists, and service users questioning its scientific legitimacy, ethical foundations, and social consequences (Adam and Juergensen 2019; Breggin 1994; Burstow 2015; Chamberlin 1978; Fabris 2011; Holmes et al. 2015; Johansson and Holmes 2023; van Daalen‐Smith 2011). Notwithstanding this, the field may be heading into existential upheaval, and while its dissolution may be difficult to predict, there is growing evidence to suggest that it might be entering an opportune paradigmatic crisis marked by intensifying resistance and epistemic instability. This crisis is not merely the result of internal professional disputes or reformist or abolitionist critique, but reflects a broader collision between psychiatric logics and emerging posthuman modes of thought that destabilize the very ontology on which psychiatry depends.

Within mental health nursing, psychiatric knowledge has long been taken as foundational, and as such, nursing practice, education, and ethics have been deeply entangled with psychiatric classification systems, risk frameworks, and treatment protocols (Adam et al. 2022; 2024; Ejaredar and Hagen 2014; Holmes and Perron 2020; van Daalen‐Smith et al. 2019). As a result, nursing is implicated not only in psychiatric care but also in the reproduction of psychiatric power (Adam 2017). This makes a critical examination of psychiatry's structural effects and nurses’ responses to them especially urgent for the discipline. Rather than asking *whether* psychiatry is simply helpful or harmful, a binary that has long polarized debate between reformist and abolitionist positions, I situate psychiatry as an inherently and structurally harmful apparatus that systematically shapes conditions of suffering, marginality, and exclusion.

A structural analysis of health/harm challenges individualist models that locate responsibility for illness/risk primarily in personal choice, behaviour, or resilience. It emphasizes how health and harm are re/produced through social, political, economic, and historical conditions. Psychiatry, when viewed this way, cannot be reduced to a neutral medical specialty responding to discrete pathologies. Rather, it functions as a distributed structure that intersects with law, policing, welfare systems, education, and the carceral state to produce and govern unruly or non‐normative forms of subjectivity (Burstow 2015; Hagen 2007; Healy 2012; Minkowitz 2014; Whitaker 2002). Mental health legislation, such as involuntary confinement and treatment laws (e.g., Ontario Mental Health Act, R.S.O 1990), exemplifies this convergence. These frameworks authorize professionals (doctors, nurses, police officers, lawyers, judges) to participate in coercive interventions justified as care. Although such interventions are often undertaken with benevolent intent, their effects are frequently violent, stripping individuals of autonomy, credibility, and bodymind sovereignty. Importantly, this harm is rendered morally invisible by its framing as therapeutic necessity. As C. S. Lewis (1970) famously warned, coercion enacted ‘for the good’ of its victims may be among the most oppressive forms of tyranny, precisely because it is sanctioned by conscience and professional ethics.

Psychiatry's structural harms are not incidental but systematic. Diagnostic practices, particularly those codified in the Diagnostic and Statistical Manual of Mental Disorders (DSM) (American Psychiatric Association [APA] 2022), operate as classificatory technologies that produce hierarchies of the human. These classifications both generate and require a psychiatric other–subjects deemed irrational, disordered, or incompetent–whose existence justifies continued psy intervention. While some individuals experience psychiatric treatment as helpful or even life‐saving, these experiences coexist with widespread evidence of coercion, overmedication, and epistemic injustice (Andre 2009; Breggin 1994; Healy 2012; Whitaker 2002). This contradiction is not accidental but intrinsic to psychiatry's structural logic (Burstow 2015).

Psychiatry's harms are unevenly distributed. A sizable body of scholarship demonstrates that psychiatric diagnosis is deeply shaped by racism, colonialism, sexism, ableism, and heteropatriarchy. Historically, psychiatry has pathologized resistance to oppression, as exemplified by diagnoses such as drapetomania, which framed enslaved Africans’ attempts at escape as mental illness (Bynum 2000). Contemporary research continues to show racialized diagnostic disparities, particularly the dramatic overdiagnosis of psychotic disorders among Black men (Metzl 2009; Schwartz and Blankenship 2014). Feminist scholars have likewise documented how psychiatry medicalizes women's responses to trauma and structural inequity, transforming anger, distress, or nonconformity into evidence of pathology (Burstow 2005; Shaw and Proctor 2005). Similarly, sexual and gender diversity (and various other ways of being such as childhood phenomenologies) have been and continue to be psychiatrized into present day. Although some diagnostic categories have since been revised or removed, the underlying medical authority and its supporting structures remain largely intact. These patterns are not relics of a bygone era but persist through psychiatry's contemporary practices and geographic expansion. The exportation of psychiatric frameworks into the Global South has been found to displace Indigenous and local understandings of distress and to reproduce colonial relations of knowledge and power (Mills 2014). Psychiatry's entanglement with global capitalism (Chapman 2023), pharmaceutical industries (Burstow 2015), and the cognate disciplines (Adam 2017) further entrenches its reach, making resistance increasingly difficult.

## 2 Critical Responses and Their Limits

In response to psychiatry's harms, a wide array of critical movements has emerged, including antipsychiatry, the psychiatric survivor movement, mad studies, neurodiversity advocacy, and identity‐based and intersectional critiques rooted in gender and disability studies, and anti‐racism and queer theory. Nursing scholars have contributed significantly to these critiques by interrogating coercive practices, foregrounding survivor knowledge, and exposing power asymmetries within psychiatric care (Adam and Juergensen 2019; Adam et al. 2022; Ejaredar and Hagen 2014; Holmes et al. 2015; Johansson and Holmes 2023; van Daalen‐Smith 2011; van Daalen‐Smith et al. 2019). These movements challenge psychiatry's claims to scientific neutrality, its reliance on diagnostic labeling, and its production of the ‘mentally ill’ as a diminished category of the human. However, many of them (some of my own earlier work included) remain tethered to a humanist framework that seeks recognition, inclusion, or rights for the mad subject. While politically vital, this strategy risks re/stabilizing the very subject psychiatry produces, leaving intact the broader ontological assumptions that authorize psychiatric governance. In this article, I argue that psychiatry operates as a humanist apparatus of capture that stabilizes the subject through diagnostic legibility, and that many critical responses–including mad identity and neurodiversity politics–paradoxically re/produce this capture by inscribing madness as a recognizable identity. My analysis is grounded in posthuman and poststructural traditions that reject the stable, coherent subject as the basis for producing knowledge. I write from an orientation that understands subjectivity as relational, contingent, and continuously in processes of becoming. This is the position I hold, however partial, perspectival, and *situated*, as Donna Haraway (1988) puts it. From this orientation, I challenge the humanist assumptions embedded in psychiatric and nursing epistemologies and approach madness as a force that resists and disrupts representational capture. Methodologically, I work within a psychiatry‐abolitionist framework–not with the expectation that I, or any individual or bounded community, could *achieve* the abolition of psychiatry, but because claims of enactment would replicate the dualistic, oppositional logics my theoretical commitments seek to unsettle. My orientation is therefore also methodological: Foregrounding instability, process, and relationality over identity, coherence, and individualized narratives. This positioning shapes the trajectory through which the arguments of this paper unfold. Drawing on posthuman (Braidotti 2011, 2013, 2019) and poststructural (Deleuze and Guattari 1983, 1987) theory, I argue that critical responses must move beyond subject‐centered humanist frameworks toward practices of becoming, imperceptibility, and connectivist thought that promise to elude psychiatry and its violent subject formation.

### 2.1 Toward Posthuman Figurations of the Mad Subject

An interrogation of the subject cannot proceed without confronting the concept of *the human* itself. Within nursing, critiques of psychiatry frequently take the form of calls to ‘humanize’ care: To render psychiatric practices ostensibly more compassionate, ethical, and responsive to lived experience (e.g., involving patients in the development of their care plan, consulting former patients on their lived experience for policy development, giving patients a right to appeal certain decisions made by the psychiatrist, etc.). While such efforts are politically and ethically motivated, they remain constrained by a deeper ontological problem. Humanization presumes the human as a stable moral and political category and assumes that those excluded from it can be restored through recognition, reform, or rights‐based inclusion. The problem is not simply that psychiatry has failed to be humane, but that the category of the human–historically and structurally–has functioned as a mechanism of exclusion, hierarchy, and capture (Braidotti 2013; Bruce 2021; Minh‐Ha 1988; Pickens 2019; Warren 2018). Nursing remains deeply invested in subject‐centred care, and this must elicit serious tensions with the humanization project, given attempts to assimilate madness into the order of the human also inadvertently reinforce the very ontological structure that renders the mad subject governable, intelligible, and capturable.

### 2.2 The Problem With the Human

The modern conceptualization of the human emerges from Renaissance Humanism, a European intellectual tradition that centred the human as the primary locus of reason, morality, and value (Law 2011). While often celebrated for its contributions to art, science, and education, Renaissance Humanism also established a normative figure of the human as *Man*–implicitly white, straight, European, male, able‐bodied, rational–who came to stand in for humanity as such. This figure was not universal but majoritarian (Deleuze and Guattari 1987), which became a standard against which all others are measured and found wanting. This majoritarian Man captures the idea that he is the molar ontological presupposition, the given and the always‐already‐there; a signifying entity that rests on the existence of a duality that locks in his other in a perpetual state of inferiority and dispossession. He speaks on behalf of the species, mediating, representing, and dis/allowing access to social goods in the name of so‐called humanity. Humanism's emphasis on autonomy, rationality, and individual moral responsibility produced an ontology that fractured the world into the well‐known Cartesian dualisms of reason/unreason, mind/body, structure/agency, nature/culture, etc. Psychiatry emerged as a primary institutional site for adjudicating these divisions, rendering those whose thinking and/or behaviour deviated from normative rationality less‐than‐human and temporarily or permanently excluding them from full subjecthood (Domingue and Foth 2025). This humanist ontology remains foundational to nursing epistemology, where even practices touted as ‘person‐centred’ often presume a coherent, rational subject beneath distress–someone who can be recovered, stabilized, or restored. However radical their spirit and vision, subject‐centered critiques remain bound to interventional frameworks through which freedom cannot emerge. What they inadvertently demonstrate with their perennial oedipalization of the subject, is that the subject may be wounded, but its structure always remains intact.

Contemporary critiques of psychiatry–including mad studies writ large–have challenged this dehumanization by reclaiming madness as an identity and standpoint. These movements have exposed epistemic injustice, foregrounded lived/living experience, and resisted psychiatric authority. However, they often do so through the politics of recognition, seeking inclusion within the category of the human rather than dismantling it altogether. This gives rise to a central paradox: Madness functions as a force that destabilizes the subject–disrupting coherence, narrative continuity, and rational self‐presence–yet it is increasingly mobilized as an identity that re‐stabilizes subjectivity. The mad subject becomes legible once more, now recognized rather than pathologized, but still situated within a humanist ontology of identification.

Human rights discourse, for example, is often activated to counter psychiatric coercion, framing involuntary treatment and confinement as violations of fundamental dignity. Yet human rights operate within the same ontology that animates psychiatric power, presuming a coherent, fully human subject, while in practice, producing a partial, semihuman whose rights exist only insofar as they are recognized and can always be rescinded. Human rights are not universally held but contingently bestowed. For those structurally excluded, rights are conditional, revocable, and unevenly prescribed. The history of psychiatry mirrors this pattern such that even when rights are formally acknowledged, they are routinely suspended in the name of care, safety, or public order (Foucault 1988). Afropessimist thinker Calvin Warren (2018) argues that the Black subject is both necessary and despised within humanist metaphysics–required to define the human yet excluded from it. He explains that whiteness is dependent on the simultaneous existence *and* annihilation of Black bodyminds, that for whiteness to exist, it needs the Black subject to exist, but perpetually kept in a state of semihumanity, occupying a class of non‐being. A similar logic operates in psychiatry, where the mad subject is simultaneously essential *and* disposable: Necessary to justify psychiatric authority yet denied full ontological legitimacy. Accordingly, psychiatry both requires the mad bodymind–indeed, existentially depends on it–and simultaneously seeks to destroy it, though only to the extent that it can be resuscitated once again, thereby rendering psychiatry necessary to ‘care’ for it anew. In both cases, liberation offered within humanist frameworks remains partial, contingent, and consequently, reversible. For the mad subject specifically, irrevocable access to human rights remains out of reach within humanist geometries of critique, given the gatekeeper effect remains fully intact long after recognition of their violation has been declared. Madness, as such, becomes coded as a defining antecedent of semihumanity, effectively rendered an implement in the psychiatric toolkit.

## 3 Recognition as Capture: Identity, Difference, and the Governable Subject

I want to state unequivocally that the most substantial gains in mental health reform have emerged from activist movements organized around identification. Mad people, psychiatric survivors, and neurodivergent communities have been steadfastly exposing coercive practices, challenging biomedical authority, and forcing institutional accountability. These efforts have reshaped research agendas, educational curricula, and policy frameworks, including those within nursing. Such work remains indispensable. However, identification also carries ontological consequences. Identity‐based politics often begin and end with the human subject, with difference serving as their organizing principle. Difference is posited as both the source of oppression and the site of emancipation, serving as the impulse for its activism. Yet, as posthuman theorists have long argued, difference has historically been overcoded as alterity. It has been poisoned and sedimented with repugnance, that “to be different from means to be worth less than” (Braidotti 2011, p. 17). Identification thus begins from a position already saturated with enduring negation. Difference, Braidotti argues, must be rethought not as divergence from a norm but as multiplicity without hierarchy. Yet identity politics frequently remain tethered to the very metaphysics they seek to undo, that is, the hegemony that spawned the ontology of difference as objectionable in the first place. The mad subject, for example, is only conditionally granted rights to its humanity directly contingent upon compliance with psychiatric norms and performativity of recovery, insight, or stability. Ontologically, the subject's humanity is never secured; its sovereignty is never recognized. It remains positioned outside full humanity, occupying a precarious zone of recognition that can be withdrawn at any moment. Insofar as the subject labels itself a product of psy oppression, the ontology of the semihuman will continue to haunt–and thwart–its efforts at seeking freedom. This should raise difficult questions for identity‐fuelled mad activism. If nursing epistemology is grounded in the humanist subject, and if that subject is itself a mechanism of intractable exclusion, then the discipline risks perpetuating the very violences it seeks to alleviate. The problem is not ethical intent but ontological inheritance.

Drawing on Deleuze and Guattari's postidentitarian framework (1987), identity can be understood as a molar formation: A stabilized, perceptible, and governable aggregation. While identity can promise belonging and safety, it also renders the subject available for capture. It brands the subject as an alter and marks it with new language–reclaimed or otherwise–that exposes it to the psychiatric oculus, in turn, risking its reterritorialization. The idea of *becoming*, in contrast, operates at the molecular level–as a process rather than a position, a line of flight rather than a category. It is attuned to the relations between humans, non‐humans, discourses, practices, etc. For nursing, this distinction is crucial. Care oriented toward identity risks reinstating the very subject psychiatry requires, whereas care oriented toward processes of becoming opens possibilities for deterritorialization, strategic illegibility, and eventually, escape.

This impasse points to a deeper problem: Not only psychiatry, but the concept of the human itself is fundamentally flawed. Posthumanist theory offers resources for grappling with this problem by rejecting human exceptionalism, dualist and hierarchal divides, and representational epistemologies. Thinkers such as Rosi Braidotti (2013; 2019) conceptualize the posthuman as both a historical condition and a theoretical figuration–a way of rethinking subjectivity as relational, processual, and more‐than‐human. In this view, subjectivity is understood as an ongoing phenomenon of *becoming*, shaped by material, discursive, and ecological entanglements.

For nursing, posthumanism opens new possibilities for understanding care, responsibility, and ethics beyond the management of bounded subjects. Rather than seeking to secure recognition for the mad subject within existing frameworks, posthuman approaches invite more radical reconfigurations of care that attend to processes, relations, and conditions rather than identities alone. In this context, psychiatry's paradigmatic crisis that I allude to in the beginning of this article can be understood not simply as institutional decline but as a symptom of a broader ontological shift. As posthuman thought gains traction, the assumptions underpinning psychiatric authority–classification, categorization, stable subjects, objective diagnosis, and concentrated governance–become untenable. Engaging this shift is central to rethinking how nursing care is conceptualized, enacted, and ethically justified in a world where the limits of both psychiatry and the human are becoming increasingly visible.

### 3.1 Nursing Critique and the Trouble With the Subject

Mental health nursing is a discipline fundamentally organized around the figure of the subject. Whether articulated as patient, person, service user, or client, nursing knowledge presupposes a human subject who is coherent, narratable, and ethically legible. This subject is often framed as distressed yet recoverable, fragmented yet ultimately capable of reintegration into normative social life. Here, I am reminded of the incarcerated patient who, often under coercive circumstances, accepts their hospital discharge under contract to be placed on community treatment orders.^2^ This orientation reflects nursing's historical inheritance from humanist philosophy, which situates the (however problematic) human subject as the primary locus of meaning, care, and moral responsibility (Adam et al. 2021). Lining up with analyses from critical movements such as the mad movement, the antipsychiatry constituency, and psychiatric survivor works, in recent decades, nursing scholarship has taken up perspectives that challenge psychiatric authority and biomedical reductionism. In that respect, these critical analyses have been mobilized to contest coercive practices, epistemic injustice, and the violence of diagnosis. While these developments mark important ethical and political interventions within nursing, they also raise a fundamental philosophical question: What kind of subject is assumed, produced, and maintained through these critiques?

Despite its oppositional stance toward psychiatry, much critical nursing scholarship–and identity‐based critical mental health critiques more broadly–continues to anchor its political project in the re/stabilization of a subject whose very coherence is produced through the same regimes it contests. Madness is mobilized as a standpoint or positionality that can be recognized, affirmed, and represented. While this resists pathologization and overt governance, it also reinstates madness within a humanist logic of subjectivity.

### 3.2 Im/Perceptibility and the Psychiatric Oculus

Psychiatric power depends on perceptibility. Diagnostic systems such as the DSM function as classificatory ontologies that render madness visible, nameable, and actionable. Classification is not neutral. It is a technology of capture that translates heterogeneous experiences into standardized categories, re/territorializing them and responding to them with clinical and carceral logics. This includes processes of appropriation of mad, survivor‐based, and activist language by the psychiatric complex–despite is insurgent reclamation. For nurses, participation in assessment, documentation, risk management, and advocacy embeds them within this classificatory regime. Even when undertaken with ethical intent, these practices stabilize the subject and reaffirm psychiatry's epistemic authority. Madness becomes legible only insofar as it conforms to existing categories, and consequently, those phenomenologies that do not fit within the psy discursive order (i.e. mad, psychiatric survivor, etc.) risk serving as content that seeks to modernize psychiatric discourse.

Deleuze and Guattari's (1987) concept of becoming‐imperceptible offers a radical counterpoint. Rather than seeking visibility or recognition, becoming‐imperceptible dissolves the coordinates that make the subject knowable, interpretable, and governable. It does not deny experience but resists its translation into any form of recognition. It ruptures out of the loculations of identity and its varied intersectional truncations and functions in a different register. This challenges deeply held assumptions about nursing advocacy, representation, and voice, suggesting alternative ethics of accompaniment, withdrawal, and relationality.

### 3.3 Nursing Beyond the Subject

Posthumanist theory furnishes possibilities for escape, offering ways to destabilize the subject in efforts to illuminate its constructed and contested nature. That the subject has come to be understood within Humanism's limited criteria is thus exposed as a construction that serves the hegemonic few. By rejecting human exceptionalism and arbitrary hierarchies, posthumanism reframes subjectivity as relational, distributed, and more‐than‐human. Cary Wolfe (2009) reminds us that what makes us ‘us’ is never really purely human but emerges from entanglements with technologies, institutions, environments, histories, and cultural inheritances that precede and exceed the individual. By way of denying supremacy to the human, nursing can also exit practices of anthropocentrism that revere a human subject constructed around the limiting characteristics of Humanism's figure of Man.

For nursing epistemology, this reframing aims to destabilize the subject as the primary unit of care. It subverts the purported enclosure of such a subject and in the process, decenters that which has come to occupy its hegemonic position. This has begun to draw attention from nurses, for example, in the work of Smith et al. (2022), which demonstrates how subject‐centered care serves only those few considered fully human, given that Humanism's subjecthood is selectively bestowed as such. In another example, Dillard‐Wright et al. (2024) discuss how nursing is already entangled with posthumanism, offering new and affirmative possibilities for how the discipline can continue to engage with human, non‐human, and more‐than‐human relations. This also holds implications for the mad‐identified subject, a point recently taken up by other nursing scholars who critique the framing of ‘the mental health crisis’ and show how the mad subject is persistently positioned within a state of non‐being (Domingue and Foth 2025). Examining the juncture of the nurse/mad‐subject dyad and drawing on posthuman and poststructural perspectives, Johansson et al. (2024) take up Deleuze and Guattari's figure of the *sorcerer*, proposing a mode of nurse‐becoming‐sorcerer that opens new possibilities for nursing practice, particularly in outreach work with mad communities. Madness, understood posthumanly, is not located within an individual bodymind but arises from assemblages of material, social, political, and institutional relations. This aligns with Deleuze and Guattari's (1987) understanding of the self as a threshold, always potentiating relations within and between multiple others–humans and non‐humans. Rather than ‘humanizing’ the mad subject (or psychiatry), this perspective invites nursing to rethink care without relying on the category of the human at all. This is a critical step in shifting towards processes of becoming.

## 4 A Thousand Tiny Madnesses

In the preceding analysis, I advocate for madness not as an identity or position to be stabilized but as a force that destabilizes the humanist subject and its attendant epistemologies. From a posthuman perspective, madness can be understood as a processual becoming, which helps jettison it out of the fixity and nominalization of identity; a sort of becoming‐mad. Becoming‐mad marks a movement away from molar identity and toward what Deleuze and Guattari call molecular proliferation: The release of ‘a thousand tiny madnesses’ (after Grosz 2018) that refuse capture by a single signifier. Within this view, becoming‐mad is both a singular strategy for evasion and a collective effort at rejecting the rigidity and immobilization of the politics of recognition. Where ‘mad’ as noun signifies an entity, a thing (*mad person*), becoming‐mad dissolves the boundaries that nominalize what is otherwise processual and simultaneously multiple. Indeed, neurodiversity is an apt example of the fracturing up of madness into an effervescent plurality of microsubjects that continue to morph, move, and for the most part, evade capture. In brief, the neurodiversity movement understands neurological differences (such as autism, ADHD, dyslexia, and others) as natural variations of human diversity rather than deficits or disorders to be cured. It advocates for acceptance, accommodation, and social change, while challenging medicalized and pathologizing frameworks that marginalize people who identify as neurodivergent. This is visible in the movement's deterritorializing interventions, that is, its rapid discursive creativity of the variety of micro‐identifications (neurodivergent, neuroqueer, neurospicy, neurocreative, and so on). This framing places contemporary neurodiversity politics in a generative but unresolved tension, including that which is generated from an assumed positionality of the ‘diverging’ subject. While neurodiversity has significantly deterritorialized psychiatric subject formation and challenged deficit‐based models of cognition, it remains entangled in the very metaphysics of subjectivity that both Humanism and psychiatry require in order to function. This tension can be understood through three paradoxes that emerge when neurodiversity is mobilized as a humanist emancipatory project within a posthuman terrain. These paradoxes do not invalidate neurodiversity activism; rather, they illuminate the limits of identity‐based resistance when the ontological ground of the subject remains intact.

### 4.1 Paradox 1: Exclusive Inclusion

The first paradox concerns inclusion that reproduces exclusion. As critics have noted, the neurodiversity movement has struggled to adequately account for race, class, gender, and sexuality, often re/producing the same hierarchies it seeks to dismantle (Russell 2020). Racialized and queer neurodivergent scholars and activists have repeatedly emphasized that neurodivergence does not operate in isolation but is always entangled with colonial, racial, and gendered regimes of power. Yet the dominant imaginaries of neurodiversity–particularly in digital and academic spaces–have been shaped by white, cisgender, middle‐class subjects (Ellis 2023; Giwa Onaiwu 2020; Lewis and Arday 2023; Logan 2020). The phenomenon of ‘aspie supremacy,’ for example, illustrates this contradiction. It refers to a distinction made between ‘low functioning’ and ‘high functioning’ autistic people, animating a clear cognitive hierarchy within the movement. Aspie supremacy demonstrates how recognition‐based politics can reinscribe the very norms (autonomy, cognition, self‐mastery, productivity) they claim to resist. Here, cognitive difference is not merely affirmed but hierarchized, reinscribing logics of superiority closely aligned with neoliberal and eugenic commitments. What appears as an inclusive counter‐discourse thus becomes a recaptured molar formation: A stabilized identity that sorts bodyminds according to economic value and moral worth. This signifies a reterritorialization of a line of flight that once emerged with emancipatory intent but becomes captured by the psychiatric apparatus. It establishes an internal stratification of the movement that secures legitimacy by reproducing the very metaphysics of hierarchy the movement ostensibly contests.

### 4.2 Paradox 2: Diagnosis Against Itself

The second paradox emerges around diagnosis–both its refusal and its strategic political uptake. Neurodiversity activism has powerfully challenged psychiatric authority through practices of self‐diagnosis, which seek to reverse the clinical gaze and its discourse (analogous to reclaiming language such as ‘madness’) and assert epistemic agency over one's own experience. At the same time, formal diagnosis remains a crucial resource for accessing education, employment, healthcare, and legal protections. This double movement–resisting diagnosis while simultaneously seeking it–reveals the ambivalence of emancipation within a diagnostic regime. The problem is not diagnosis per se, but the ontological work it performs. Diagnostic categories stabilize experience into legible forms, anchoring difference within psy‐discursive territory. Even self‐diagnosis, when articulated through DSM nomenclature, risks reproducing the same epistemic capture it seeks to escape. Moreover, *diagnosis* as noun carries a heavy medical‐ideological charge, reanimating binaries of sick/well and normal/abnormal that firmly moor the neurodivergent subject in psychiatric discourse. Lastly and relatedly, the increasing reliance on the prefix *neuro* further entrenches this dynamic. While intended to naturalize diversity, it simultaneously reinscribes biomedical authority, tethering difference to neurological explanation and inviting biopsychiatric governance. As Žižek (2023) reminds us, language is never neutral; it carries with it a sedimented metaphysics that shapes what can be thought, claimed, or resisted. For nursing, this paradox raises critical questions about advocacy practices that rely on diagnostic legitimation: Which ontologies are rendered legitimate and at what cost to the pursuit of ontological diversity?

### 4.3 Paradox 3: Access to What, Exactly?

The third paradox concerns access–particularly ‘access’ to capitalist institutions. Neurodiversity‐informed initiatives increasingly emphasize workplace accommodations and the economic value of cognitive difference, often under the banner of neurofuturism. Papadopoulos (2026) explains that neurofuturism demands the active participation of neurodivergent people in the production of their futures, positioning neurodivergent cognitive orientations as generative resources for engaging and reshaping sociotechnical worlds. While such efforts may alleviate individual exclusion, they frequently leave institutional structures unchanged. Access is granted to the neurodivergent subject precisely insofar as they can be rendered productive within existing regimes of value (e.g., high functioning). As Chapman (2023) has shown, capitalism's ‘empire of normality’ does not merely exclude difference, it actively produces it as deviation from a financialized norm calibrated to efficiency, productivity, and control. The celebration of neurodivergent ‘talent’ risks transforming cognitive difference into a niche labour resource, folded seamlessly into what Moulier Boutang (2011) terms cognitive capitalism. Here, knowledge itself becomes the primary site of extraction, and neurodivergence is re/valued only insofar as it can be instrumentalized. This paradox underscores a deeper continuity between Humanism and capitalism. Long before bodyminds were rendered as productive machines, Humanism had already stratified the subject according to reason, ability, and worth. Capitalism merely intensified this logic. The danger for nursing lies in conflating access with justice and accommodation with transformation, thereby mistaking integration into oppressive systems for emancipation from them.

Across these paradoxes, a common thread emerges: Resistance that begins and ends with identity risks reinscribing the metaphysical architecture of the subject and its humanist baggage. Neurodiversity, when mobilized as identity, may secure recognition but often at the expense of ontological disruption. A posthuman approach suggests a different horizon–one oriented not toward stabilizing who the neurodivergent subject *is*, but toward multiplying how *becoming* can occur. Finally, I also want to emphasize that the movement is already, to some degree, working within postidentitarian framings. Chapman (2023), for example, deterritorializes ‘neurodivergent identity’ by shifting from recognition politics to the machinics of capitalism's empire of normality: The problem is not who we are, but how apparatuses of productivity, value, and normation stratify bodies and brains into legible types. He swaps identity for diagram–tracking, via historical materialism, how assemblages produce ‘normality’ as a capture‐device–and calls for lines of flight that recompose relations at the level of material flows, labour, and capacity rather than stable categories. Put simply: Not identities demanding inclusion, but transversal reconfigurations that short‐circuit the regime of normality and open space for new compositions of life.

## 5 Captured by Freedom: Identity, Madness, and Posthuman Rupture

The aphorism attributed to Johann Wolfgang von Goethe, *none are more hopelessly enslaved than those who falsely believe they are free*–offers a productive starting point for thinking about madness, psychiatry, and resistance. The force of the statement lies not in moral judgment but in its diagnosis of power: Freedom is not negated solely by overt domination but by forms of capture that masquerade as liberation. For instance, a psychiatric patient permitted to leave the hospital on a pass experiences little meaningful freedom. Departure is contingent on being signed out by a family member or friend, and movement remains tightly regulated through mandatory return times that reproduce institutional control in the guise of leniency.

This insight resonates with the paradoxical position of mad identities, which have simultaneously enabled resistance to psychiatric violence and risked reinforcing the very hegemonic structures they seek to escape. Minh‐Ha (1988) explains:

Hegemony works at leveling out differences and at standardizing contexts and expectations in the smallest details of our daily lives. Uncovering this level of difference is, therefore, resisting that very notion of difference which defined in the master's terms, often resorts to the simplicity of essences.

(p. 2)

This is what can–and indeed often does–lead to the molarization of the subject and its lived/living experience, even within some of the most radical movements. Just when one establishes itself and locks in an identity, it inherently begins to operate in exclusion of subjectivities who might be parallel to its cause but fail to neatly fit into its ontological enclosure. It is a question of us/not‐us or in/out, as Sumner (1906) would have theorized over a century ago, a perennial dualist ontology that continues to haunt anti‐oppression political movements and their associated activist efforts. It is an ontology of recognition that locates the othered subject (I) as an essentialized capsule, continuously furnishing conditions to generate a secondary other (not‐I), triggering the production of exteriorized locules of rejected identities. There is also the issue of madness as a received identity, handed down, bestowed upon those whom to various degrees might reject it. In this context, it is superimposed, laid over the subject's consciousness. As such, those whose madness is a source of intractable misery have no recourse to *become* otherwise, with little choice but to assimilate into the subjectivity of the ‘mad,’ while marching along in allegiance to a dialectic of I/not‐I that distills differences, creativities, and affirmations into various genres of the ‘oppressed.’ Drawing on gender as an example, Minh‐Ha (1988) continues:

How am I to lose, maintain, or gain a female identity when it is impossible for me to take up a position outside this identity from which I presumably reach in and feel for it? Difference in such a context is that which undermines the very idea of identity, differing to infinity the layers of totality that forms I.

(p. 2)

The animation of any identity or standpoint depends upon exclusion. Identity operates through this logic of capture, producing in‐groups and–by extension–out‐groups. That an immediate, peripheral other must exist in simultaneity with the formation of an identity becomes a paradoxical process of lateral othering that begins to undo itself, acting as its own antithesis. In seeking freedom, the identity‐bound subject instead confines itself to what is perceptible and capturable, while either assimilating its excluded lateral other or banishing it to a zone reserved for those who fail to meet its criteria for inclusion. Freedom, in this sense, is reduced to an illusion, an endlessly pursued mirage.

## 6 Final Words: Away With the Human

I have argued that psychiatry functions as a structural apparatus of capture predicated on the stability and stabilization of the subject qua identity. Madness as *becoming* operates as a force of instability–one that unsettles coherence, challenges representation, and resists reduction to identity. Yet the political necessity of organizing against psychiatric harm has often required recourse to identification: Naming oneself as mad, neurodivergent, or a psychiatric survivor in order to claim recognition, rights, and voice. This gives rise to a central paradox that demands sustained philosophical attention within nursing: Identity is both liberatory and entrapping. This paradox can be theorized as a form of double capture. The first capture occurs when madness is apprehended by the psychiatric apparatus–classified, diagnosed, and rendered legible through psy discourse. The second capture follows when madness is reclaimed as identity by way of reverse‐discourse. While this second capture is often undertaken strategically and with emancipatory intent, it nevertheless stabilizes madness within a recognizable form, rendering it once again perceptible and governable. From a posthuman perspective, this reliance on the subject is itself the problem. Psychiatry does not merely fail to represent madness adequately; its vocabulary is structurally incapable of doing so. The contradictions, intensities, and molecular variations of becoming‐mad exceed representational capture. Psychiatry's failure is not accidental but constitutive. Its discursive limits are precisely what allow it to function as a technology of co‐optation. It is at these limits where the processing of madness and its appropriation occurs, reterritorializing it into reformed psy vernacular.

For far too long, the nursing discipline has nurtured a violent human, an ethical contamination striated by the suffering of bodyminds denied any claim to humanity. As this human comes to represent the species, he signifies, individuates, and divides. He is the molar aggregate who disaggregates. His primary objective is to render an other as a negative image of himself and maintain a dialectical antagonism with it in the service of his own thriving. He generates separations of humanness, re/territorializing them into hierarchies that sustain his apparatuses of symbolic and material capture and violence. Against this backdrop, the question is not whether to resist psychiatry, but how to do so without reproducing its ontological conditions and nurture its humanist molarity. Deleuze and Guattari offer a conceptual vocabulary for thinking about escape beyond opposition–both for the so‐called mad subject and the critical efforts deployed in the service of its emancipation. They offer the idea of becoming‐imperceptible by forging lines of flight from/within/through humanist forms of subjectivity–creating avenues of rupture that move alongside and/or outside binary notions of subject/object, in/out, etc. In this sense, escape and capture are not dual opposites but overlapping flows. The task, however, is not to abolish identity^3^ outright but to loosen its grip, to move through it without becoming fixed and/or represented, and to decenter Man and his dualist hierarchies. Forming lines of flight and becoming‐imperceptible does not entail disappearance or silence. It names a postdual, postidentitarian mode of existence that evades recognition and stability, even if fragile and fleeting. It is a refusal of symbolic availability, a way of operating below or beyond the perceptual radar of regimes that enable governance. This move offers a model for a processual mad subject for nursing: Itinerant, transversal, enigmatic, and resistant to capture. It names a form of resistance that does not seek recognition, reform, or integration but invents new ways of living that psychiatry cannot easily appropriate.

To engage madness posthumanly is to rethink nursing epistemology and care beyond the human altogether. It also demands caution, given that lines of flight are always at risk for recapture (Deleuze and Guattari 1987). Collaborative initiatives that expand psychiatric reach under the banner of inclusion or consultation may inadvertently strengthen the psy apparatus. In her antipsychiatry ‘model of attrition,’ for example, Burstow (2014) similarly cautions that insurgent strategies may backfire, inadvertently expanding psychiatry's reach and capacity for capture. An ethics of becoming‐imperceptible does not abandon care but reconfigures it. It privileges accompaniment over assessment, support over surveillance, and relation over recognition. It also acknowledges limits. There is no absolute freedom, only lines and segments of fragile escape. Becoming‐imperceptible unfolds as its own process, wherein imperceptibility–however momentarily attained–can never be guaranteed and is always threatened by the forces of psy reterritorialization. Consequently, nurses must be cautious. Structures of control may erode, but they reconstitute themselves in new forms that seek to construct and govern new subjectivities, hence the impossibility of fully escaping the subject. Accepting this paradox need not lead to resignation. Rather, it invites a more sustainable orientation toward struggle–one that values moments of fugitivity, pockets of opacity, and collective intensities.

While becoming‐imperceptible offers a sleek line of flight from psychiatric capture, a hard objection might surface here: For those already rendered unseen, defunded, and left to weather institutional withdrawal–disproportionately racialized, poor, and multiply marginalized–an ethic of imperceptibility risks dressing up abandonment as liberation. I want to underscore a crucial distinction between becoming‐imperceptible and being rendered *structurally invisible*. Becoming‐imperceptible is neither a disappearance nor a retreat into concealment; it is a mode of sub‐radar movement–an active, tactical practice of recalibration, strategizing, and evasion. It involves slipping just out of phase, introducing a subtle dissonance in tempo that opens a brief but generative interval. In this sense, it is a retiming of the subject in relation to the apparatus: A deliberate desynchronization that scrambles the cycles through which capture anticipates, recognizes, and secures its objects. I argue that becoming‐imperceptible functions, in part, as an antidote to structural oppression and enforced invisibility. To be rendered voiceless, negligible, or erased from political view on grounds of race, gender, sexuality, disability, or class is itself a form of capture–in many ways, *capture at its most perfected*. Within this regime, the marginalized subject does not simply disappear; rather, they are held in place by the very mechanisms that claim not to see them. Thus, for those already consigned to institutional invisibility, becoming‐imperceptible is not an intensification of disappearance but a counter‐maneuver: A way to push back against imposed erasure by resurfacing differently, tactically, and only to the extent necessary to sustain life within the wavelength of imperceptibility. It is a movement that allows one to endure and improvise–to remain partially out of reach while still asserting a presence that resists annihilation. In this light, the two conditions are not only distinct but incompatible. Structural invisibility is a violence, a mode of capture, whereas becoming‐imperceptible–understood not as vanishing but as affirmative misalignment–operates as a practice for interrupting, rerouting, and redressing the very violences imposed by institutional erasure.

Positioned at the intersection of care and governance, nursing holds a crucial role in cultivating these practices without claiming ownership over them. In animating affirmative possibilities for care, María Puig de la Bellacasa (2017) asserts that care is not solely a human‐centered moral practice but an ethical, political, and material engagement that extends across more‐than‐human worlds. Puig de la Bellacasa argues that to understand care in contemporary ecological and technoscientific contexts, it must be rethought as an entangled practice involving humans, nonhumans, technologies, environments, and sociotechnical systems. In this register, rethinking nursing care–including not only psychiatric care but also the forms of nursing practice that resist and work against dominant mental health paradigms across hospitals, clinics, prisons, patient homes, and elsewhere–requires a shift in how care itself is conceptualized. What emerges when nurses understand care not as something done *to* or *for* a bounded individual subject, but as something generated through assemblages of bodies, spaces, affects, institutional arrangements, and more‐than‐human forces? How might nurses offer care within moments of tension or paradox that destabilize the fundamental ontological assumptions of the discipline? Puig de la Bellacasa's insight is informative here. Affirmative possibilities exist, but they are exceedingly limited, and because they arise outside clinical and institutional spaces, remain somewhat distant from most nursing practice. Examples such as peer respites, intentional peer support, peer‐led mutual aid networks, community‐based crisis houses, and peer‐run wellness programs offer current non‐medical alternatives to psychiatric care and signal lines of flight in an increasingly postpsychiatric world. These modes of care signify a resistance to humanist ontologies that currently substruct psychiatric logic, demonstrating a rupture within its molar architecture. I want to remain non‐prescriptive, proposing only this minor gesture: Attune nursing to already‐effective resources that move rhizomatically across worlds, and, within each field of care, advocate and model their immanent repetition.

The paradox of identity–its capacity to both liberate *and* capture–cannot be resolved once and for all. It must be lived with, negotiated, and strategically deployed. When understood as a process of *becoming* rather than an identity, madness can offer a site from which to unsettle the humanist subject and its signifying psychiatric enclosures. For nursing philosophy, the challenge is to re/think without reinscribing the subject as the primary unit of intervention. This entails engaging with posthuman ontologies, cultivating practices of imperceptibility, and aligning with assemblages that operate outside the logic of recognition and its capture. The goal is not to abolish psychiatry through confrontation, but to render it increasingly irrelevant by proliferating forms of posthuman existence it cannot recognize, classify, or govern. Destabilizing the subject requires more than recognition, rights, or reform. It requires letting go of the human as a central organizing principle. Becoming‐mad, in this sense, is not an identity to be secured but a transversal force, a swarm of intensities, a threshold that exposes the limits of Humanism itself. For nursing, the challenge is not to perfect the human subject, but to learn how to care amid its instability. Becoming‐mad and becoming‐imperceptible do not promise freedom–from psychiatry or the subject. They offer something more precarious and more powerful: Escape.

## Ethics Statement

The author has nothing to report.

## Conflicts of Interest

The author declares no conflicts of interest.

## Data Availability

Data sharing not applicable to this article as no datasets were generated or analyzed during the current study.

## Endnotes

2 A *community treatment order* is a legal directive issued by a doctor that obligates a person to receive supervised psychiatric treatment while living in the community. Positioned as a less restrictive option than inpatient detention, it nonetheless extends coercive governance into everyday life by binding individuals to treatment and surveillance regimes outside hospital settings.

3 I do not believe it is possible to abolish identity as it is so closely imbricated within notions of the subject. Given that subjects are always an effect of power (following Foucault)–that power needs something to act upon–their abolition is theoretically untenable. What might be possible, however, is reframing thinking around the boundedness and identifiability of the subject, freeing it from the violent dialectic it has been mired in with Humanism's molar Man.

## References

[nup70079-bib-0001] Adam, S. 2017. “Crazy Making: The Institutional Relations of Undergraduate Nursing in the Reproduction of Biomedical Psychiatry.” International Journal of Nursing Education Scholarship 14, no. 1: 20170071. 10.1515/ijnes-2017-0071. 29240556

[nup70079-bib-0002] Adam, S. , E. Gold , and B. Burstow . 2022. “From Subjective Opinion to Medical Fact: A Critical Discourse Analysis of Mental Health Nursing Education.” Issues in Mental Health Nursing 44, no. 1: 55–63. 10.1080/01612840.2022.2113940. 35994049

[nup70079-bib-0003] Adam, S. , C. Jiang , M. Mikhail , and L. Juergensen . 2024. “Infrahuman Madness: Mental Health Nursing and the Discursive Production of Alterity.” Nursing Inquiry 31, no. 1: e12533. 10.1111/nin.12533. 36317296

[nup70079-bib-0004] Adam, S. , and L. Juergensen . 2019. “Toward Critical Thinking as a Virtue: The Case of Mental Health Nursing Education.” Nurse Education in Practice 38: 138–144. 10.1016/j.nepr.2019.06.006. 31279924

[nup70079-bib-0005] Adam, S. , L. Juergensen , and C. Mallette . 2021. “Harnessing the Power to Bridge Different Worlds: An Introduction to Posthumanism as a Philosophical Perspective for the Discipline.” Nursing Philosophy 22, no. 1: e12362. 10.1111/nup.12362. 34157215

[nup70079-bib-0006] Andre, L. 2009. Doctors of Deception: What They Don't Want You to Know About Shock Treatment. Rutgers University Press.

[nup70079-bib-0007] APA . 2022. Diagnostic And Statistical Manual of Mental Disorders, Fifth Edition, Text Revision. APA.

[nup70079-bib-0008] Braidotti, R. 2011. Nomadic Theory: The Portable Rosi Braidotti. Columbia University Press.

[nup70079-bib-0009] Braidotti, R. 2013. The Posthuman. Polity.

[nup70079-bib-0010] Braidotti, R. 2019. Posthuman Knowledge. Polity.

[nup70079-bib-0011] Breggin, P. 1994. Toxic Psychiatry: Why Therapy, Empathy, and Love Must Replace the Drugs, Electroshock, snd Biomedical Theories Of The “New Psychiatry.”. St. Martin's Press.

[nup70079-bib-0012] Bruce, L. J. 2021. How to Go Mad Without Losing Your Mind: Madness and Black Radical Creativity. Duke University Press.

[nup70079-bib-0013] Burstow, B. 2005. “A Critique of Posttraumatic Stress Disorder and the DSM.” Journal of Humanistic Psychology 45, no. 4: 429–445. 10.1177/0022167805280265.

[nup70079-bib-0014] Burstow, B. 2015. Psychiatry and the Business of Madness: An Ethical and Epistemological Accounting. Palgrave Macmillan.

[nup70079-bib-0015] Burstow, B. 2014. “The Withering Away of Psychiatry: An Attrition Model for Antipsychiatry.” In Psychiatry Disrupted: Theorizing Resistance and Crafting the (R)Evolution, edited by B. Burstow , B. LeFrançois , and S. Diamond , 34–51. McGill‐Queen's University Press.

[nup70079-bib-0016] Bynum, B. 2000. “Discarded Diagnoses.” Lancet 356, no. 9241: 1615. 10.1016/S0140-6736(05)74468-8. 11075805

[nup70079-bib-0017] Chamberlin, J. 1978. On Our Own: Patient Controlled Alternatives to the Mental Health System. Haworth Press.

[nup70079-bib-0018] Chapman, R. 2023. Empire of Normality: Neurodiversity and Capitalism. Pluto Press.

[nup70079-bib-0063] van Daalen‐Smith, C. 2011. “Waiting for Oblivion: Women's Experiences With Electroshock.” Issues in Mental Health Nursing 32: 457–472. 10.3109/01612840.2011.583810. 21736469

[nup70079-bib-0019] van Daalen‐Smith, C. , S. Adam , F. Hassim , and F. Santerre . 2019. “A World of Indifference/Un Monde D'indifférence: Canadian Women's Experiences of Psychiatric Hospitalization/Expériences Canadiennes d'hospitalization Psychiatrique Au Canada.” Issues in Mental Health Nursing 41, no. 4: 315–327. 10.1080/01612840.2019.1661047. 31770058

[nup70079-bib-0020] Deleuze, G. , and F. Guattari 1983. On the line. Semiotext(e).

[nup70079-bib-0021] Deleuze, G. , and F. Guattari . 1987. A Thousand plateaus: Capitalism and Schizophrenia. University of Minnesota Press.

[nup70079-bib-0022] Dillard‐Wright, J. , J. B. Smith , J. Hopkins‐Walsh , E. Willis , B. B. Brown , and E. C. Tedjasukmana . 2024. “Notes on [Post]Human Nursing: What It Might Be, What It Is Not.” Nursing Inquiry 31, no. 1: e12562. 10.1111/nin.12562. 37211658

[nup70079-bib-0023] Domingue, J.‐L. , and T. Foth . 2025. “The ‘Mental Health Crisis’ and the Nonbeing of the Mad.” Nursing Philosophy 27, no. 1: e70055. 10.1111/nup.70055. PMC12662625 41313796

[nup70079-bib-0024] Ejaredar, M. , and B. Hagen . 2014. “I Was Told It Restarts Your Brain: Knowledge, Power, and Women's Experiences of Ect.” Journal of Mental Health 23, no. 1: 31–37. 10.3109/09638237.2013.841870. 24484190

[nup70079-bib-0025] Ellis, J. 2023. “Imagining Neurodivergent Futures From the Belly of the Identity Machine: Neurodiversity, Biosociality, and Strategic Essentialism.” Autism in Adulthood 5, no. 3: 225–235. 10.1089/aut.2021.0075. 37663441 PMC10468558

[nup70079-bib-0026] Fabris, E. 2011. TranquiL Prisons: Chemical Incarceration Under Community Treatment Orders. University of Toronto Press.

[nup70079-bib-0027] Foucault, M. 1988. Madness & Civilization: A History of Insanity in the Age of Reason. Vintage.

[nup70079-bib-0028] Giwa Onaiwu, M. 2020. “I, Too, Sing Neurodiversity.” Ought: The Journal of Autistic Culture 2, no. 1: 10. 10.9707/2833-1508.1048.

[nup70079-bib-0029] Grosz, E. 2018. “A thousand tiny sexes: Feminism and rhizomatics.” In Gilles Deleuze and the Theater of Philosophy, edited by C. Boundas and D. Olkowski , 187–210. Routledge.

[nup70079-bib-0030] Hagen, B. 2007. “Measuring Melancholy: A Critique of the Beck Depression Inventory and Its Use in Mental Health Nursing.” International Journal of Mental Health Nursing 16: 108–115. 10.1111/j.1447-0349.2007.00453.x. 17348961

[nup70079-bib-0031] Haraway, D. 1988. “Situated Knowledges: The Science Question in Feminism and the Privilege of Partial Perspective.” Feminist Studies 14, no. 3: 575–599. 10.2307/3178066.

[nup70079-bib-0032] Healy, D. 2012. Pharmageddon. University of California Press.

[nup70079-bib-0033] Holmes, D. , S. J. Murray , and N. Knack . 2015. “Experiencing Seclusion in a Forensic Psychiatric Setting: A Phenomenological Study.” Journal of Forensic Nursing 11, no. 4: 200–213. 10.1097/jfn.0000000000000088. 26457901

[nup70079-bib-0034] Holmes, D. , and A. Perron . 2020. “Power, Discourse and Resistance in Mental Health Care.” Witness: The Canadian Journal of Critical Nursing Discourse 2, no. 2: 1–2. 10.25071/2291-5796.85.

[nup70079-bib-0035] Johansson, J. A. , and D. Holmes . 2023. “Poststructuralism and the Construction of Subjectivities in Forensic Mental Health: Opportunities for Resistance.” Nursing Philosophy 25, no. 1: e12440. 10.1111/nup.12440. 37070337

[nup70079-bib-0036] Johansson, J. A. , P.‐L. Turcotte , and D. Holmes . 2024. “Assembling Packs: Outreach Nurses, Disaffiliated Persons, and Sorcerers.” Nursing Philosophy 25, no. 3: e12486. 10.1111/nup.12486. 38853432

[nup70079-bib-0038] Law, S. 2011. Humanism: A Very Short Introduction. Oxford University Press.

[nup70079-bib-0041] Lewis, C. 1970. God in the Dock: Essays on Theology and Ethics. W. B. Eerdmans Publishing.

[nup70079-bib-0042] Lewis, C. J. , and J. Arday . 2023. “We'll See Things They'll Never See: Sociological Reflections on Race, Neurodiversity and Higher Education.” Sociological Review 71, no. 6: 1299–1321. 10.1177/00380261231184357.

[nup70079-bib-0043] Logan, J. 2020. “Queer and Neurodivergent Identity Production Within the Social Media Panopticon.” Macksey Journal 1, no. 1: 177. https://mackseyjournal.scholasticahq.com/article/21928‐queer‐and‐neurodivergent‐identityproduction‐within‐the‐social‐media‐panopticon.

[nup70079-bib-0044] Metzl, J. M. 2009. The Protest Psychosis: How Schizophrenia Became a Black Disease. Beacon Press.

[nup70079-bib-0045] Mills, C. 2014. Decolonizing Global Mental Health: The Psychiatrization of the Majority World. Routledge.

[nup70079-bib-0046] Minh‐Ha, T. 1988. “Not You/Like You: Post‐Colonial Women and the Interlocking Questions of Identity and Difference.” Inscriptions 3–4: 1–6. https://www.nonknowledge.org/media/pages/hfbksose21/911581847‐1621940841/trinh‐t.‐minh‐ha‐not‐you‐like‐you.pdf.

[nup70079-bib-0047] Minkowitz, T. 2014. “Convention on The Rights of Persons With Disabilities and Liberation From Psychiatric Oppression.” In Psychiatry Disrupted: Theorizing Resistance and Crafting the (R)Evolution, edited by B. Burstow , B. LeFrançois , and S. Diamond , 129–144. McGill‐Queen's University Press.

[nup70079-bib-0048] Moulier Boutang, Y. 2011. Cognitive Capitalism. Polity.

[nup70079-bib-0049] Ontario Mental Health Act, R.S.O . 1990, c. M.7 (Can.). https://www.ontario.ca/laws/statute/90m07.

[nup70079-bib-0050] Papadopoulos, C. 2026. Voices of Neurodiversity: An Inclusive Encyclopedia. Routledge.

[nup70079-bib-0051] Pickens, T. A. 2019. Black Madness::mad Blackness. Duke University Press.

[nup70079-bib-0052] Puig de la Bellacasa, M. 2017. Matters of Care: Speculative Ethics in More Than Human Worlds. University of Minnesota Press.

[nup70079-bib-0053] Russell, G. 2020. “Critiques of the Neurodiversity Movement.” In Autistic Community and the Neurodiversity Movement, edited by S. Kapp , 287–303. Palgrave Macmillan.

[nup70079-bib-0054] Schwartz, R. C. , and M. Blankenship . 2014. “Racial Disparities in Psychotic Disorder Diagnosis: A Review of Empirical Literature.” World Journal of Psychiatry 4, no. 4: 133–140. 10.5498/wjp.v4.i4.133. 25540728 PMC4274585

[nup70079-bib-0056] Shaw, C. , and G. Proctor . 2005. “Women at the Margins: A Critique of the Diagnosis of Borderline Personality Disorder.” Feminism & psychology 15, no. 4: 483–490. 10.1177/0959-353505057620.

[nup70079-bib-0057] Smith, J. B. , E.‐M. Willis , and J. Hopkins‐Walsh . 2022. “What Does Person‐Centred Care Mean, If You Weren't Considered a Person Anyway: An Engagement With Person‐Centred Care and Black, Queer, Feminist, and Posthuman Approaches.” Nursing Philosophy 23, no. 3: e12401. 10.1111/nup.12401. 35749609

[nup70079-bib-0058] Sumner, W. G. 1906. Folkways: A Study of Mores, Manners, Customs and Morals. Ginn and Company.

[nup70079-bib-0059] Warren, C. 2018. Ontological Terror: Blackness, Nihilism, and Emancipation. Duke University Press.

[nup70079-bib-0060] Whitaker, R. 2002. Mad in America: Bad Science, Bad Medicine, and the Enduring Mistreatment of the Mentally ill. Basic Books. 12145564

[nup70079-bib-0061] Wolfe, C. 2009. What Is Posthumanism? University of Minnesota Press.

[nup70079-bib-0062] Žižek, S. 2023. Freedom: A Disease Without Cure. Bloomsbury.

